# Supplementary material for: Plasmons in the van der Waals charge-density-wave material 2H-TaSe2
Source: Nat Commun. 2021 Jan 15;12:386. doi: 10.1038/s41467-020-20720-0 (PMC7810790; doi:10.1038/s41467-020-20720-0)
Supplement: Supplementary file 1 — Supplementary Information [file 41467_2020_20720_MOESM1_ESM.pdf]

Supplementary Information for

Plasmons in the van der Waals charge-density-wave material 2H-$TaSe_2$

Chaoyu Song et al.

Supplementary note 1: $TaSe_2$ plasmons at far-IR and terahertz regions

Supplementary note 2: Simulations of the screening effect of interband transitions

Supplementary note 3: $TaSe_2$ plasmons with different film thickness

Supplementary note 4: $TaSe_2$ plasmons in different dielectric environments

Supplementary note 5: The temperature evolution of the intrinsic optical response and plasmons of 2H-$TaSe_2$

Supplementary note 6: The plasmon dispersion of 2H-$NbSe_2$

Supplementary note 7: The temperature evolution of the intrinsic optical response and plasmons of 2H-$NbSe_2$

Supplementary note 8: The coupled-oscillator model

Supplementary note 9: The Raman spectroscopy of 2H- $TaSe_2$ and $NbSe_2$ single crystals

Supplementary note 10: The ambient stability

Supplementary note 11: Carrier density determination from Hall resistance measurements

Supplementary Table 1: Summary of samples

Supplementary note 1: $TaSe_2$ plasmons at far-IR and terahertz regions

For $TaSe_2$ plasmonic devices (d = 40 nm) with ribbon width larger than 4 μm, the plasmon resonance redshifts to the far-IR and terahertz regions. Supplementary Fig. 1a shows the extinction spectra of them. The maximal light wavelength is approximately 40 μm (frequency 250 cm^{-1}) for a plasmonic device with ribbon width of 9 μm, as shown in Supplementary Fig. 1b. The plasmon peak width is about 100 cm^{-1}. The first order plasmon peak at the far-IR range continuously becomes sharper as the temperature decreases (Supplementary Fig. 1c), because it is away from the CDW excitation at the mid-IR range.

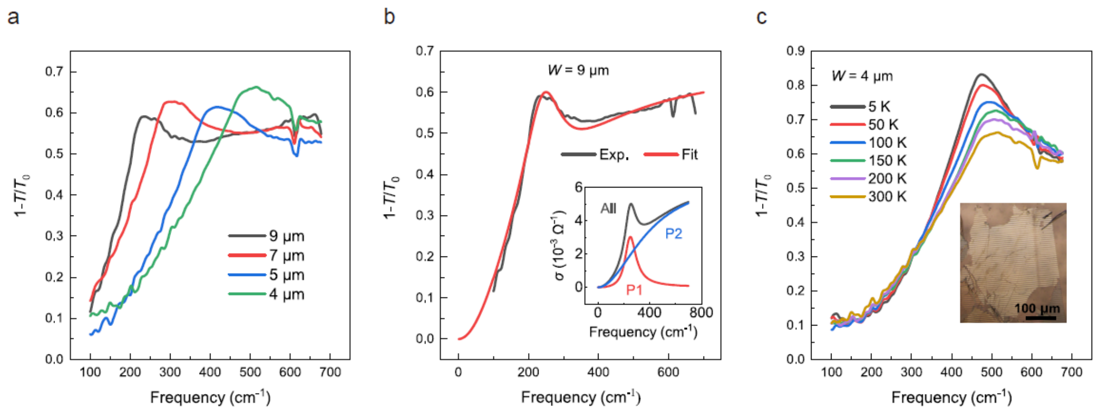

Supplementary Figure 1 Plasmons of 2H-$TaSe_2$ at far-IR and terahertz regions (a) The extinction spectra of 2H-$TaSe_2$ plasmon devices (sample TF1-TF4, d = 40 nm, W = 4-9 μm) at room temperature. **(b)** The extinction spectrum of a plasmonic device (sample TF4, W = 9 μm), the black and red solid lines are the experimental and the fitted spectra respectively. The inset shows the fitted optical conductivity, P1 and P2 correspond to the first and the second order modes respectively. **(c)** The temperature-dependent extinction spectra of a plasmonic device (sample TF1, W = 4 μm), the inset shows its optical image.

Supplementary note 2: Simulations of the screening effect of interband transitions

The plasmon resonance can be simulated by the loss function $-\mathrm{Im}(\frac{1}{\varepsilon(q,\omega)})$, which is the imaginary part of the inverse of the dielectric function[1]. The $\varepsilon(q,\omega)$ of 2D systems can be expressed as follows:

$$\varepsilon(q,\omega)=\varepsilon_{env}+\frac{i\sigma(\omega)}{\varepsilon_0\omega}\frac{q}{2} \qquad (1)$$

Where ε_{env} is the dielectric constant of the environment, which is $(1+\varepsilon_s)/2$ in our case, ε_0 is the vacuum dielectric constant and q denotes the wave vector. The sheet optical conductivity $\sigma(\omega)$, by taking into account both intraband and interband transitions, is given by:

$$\sigma(\omega)=i\frac{D}{\pi}\frac{1}{\omega+i\gamma_D}+i\sum_n\frac{S_n}{\pi}\frac{\omega}{\omega^2-\omega_n^2+i\gamma_n\omega} \qquad (2)$$

where S_n, ω_n and γ_n represent the spectral weight, the frequency and the damping rate of the n_{th} interband transition resonance, respectively. D and γ_D are the Drude weight and scattering rate, respectively. The Drude weight is related to the properties of free carriers as $D=\frac{\pi e^2 n_s}{m}$, n_s is the sheet carrier density, *m* is the effective mass. Since the frequencies of the interband transitions of metallic TMDCs are much larger than the plasmon frequency at the infrared region[2-4], Eq. (2) can be simplified as follows:

$$\sigma(\omega)=i\frac{D}{\pi}\frac{1}{\omega+i\gamma_D}-i\sum_n\frac{S_n}{\pi}\frac{\omega}{\omega_n^2} \qquad (3)$$

Then, by substituting Eq. (3) into Eq. (1), one can obtain the *q*-dependent dielectric constant of interband transitions:

$$\varepsilon_{inter}(q,\omega)=\frac{d}{2}\sum_n\frac{S_n/d}{\pi\omega_n^2\varepsilon_0}\cdot q=\frac{d\varepsilon}{2}\cdot q \qquad (4)$$

Here $\varepsilon \equiv \sum_n \frac{S_n / d}{\pi \omega_n^2 \varepsilon_0}$ effectively equals to the intrinsic dielectric constant due to the high energy interband transitions discussed in the main text. Now, we can use the loss function to simulate the plasmon resonance of the 40-nm $TaSe_2$ (sample T1). It should be noted that only the first order plasmon is considered in this model. The Drude weight D = 124 $\Omega^{-1}s^{-1}$ and Drude scattering rate γ_D = 2000 cm^{-1} are used in simulations. As illustrated in Supplementary Fig. 2a-d, the plasmon frequency prominently decreases and the plasmon dispersion at large wave vectors becomes flat as ε increases. The plasmon dispersion of sample T1 is well simulated by setting ε = 4, which agrees with the value fitted by the plasmon dispersion. In addition, the intensity of the plasmon resonance reduces as the screening effect increases, especially for that at large wave vectors.

When 2D electron systems are fabricated into disks, the parameters of localized plasmons can be analytically solved, which provides another way to analyze the screening effect of interband transitions. The optical conductivity of metallic disk arrays is given by[5, 6]:

$$\sigma_{disk}(\omega) = f \frac{\sigma_{film}}{1 - \frac{\pi \sigma_{film}}{4 a \varepsilon_0 \varepsilon_{env} i \omega}} \qquad (5)$$

f is the filling factor of the disk array, a is the disk radius and σ_{film} is the optical conductivity of unpatterned films. To better manifest the screening effect of interband transitions, we incorporate the intrinsic dielectric constant ε into Eq. (3). Then, by substituting Eq. (3) into Eq. (5), $\sigma_{disk}(\omega)$ becomes:

$$\sigma_{disk}(\omega) = \frac{f}{(1 + \frac{3\pi\varepsilon}{16\varepsilon_{env}} \frac{d}{a})} \frac{i\omega\left[\mathrm{D}/\pi - \varepsilon\varepsilon_0 d\omega(\omega + i\gamma_{\mathrm{D}})\right]}{\left[\omega^2 - \omega_{p0}^2 / (1 + \frac{3\pi\varepsilon}{16\varepsilon_{env}} \frac{d}{a}) + i\gamma_{\mathrm{D}}\omega\right]} \qquad (6)$$

where $\omega_{P0}=\sqrt{\frac{3\pi n_s e^2}{16ma\varepsilon_0\varepsilon_{env}}}=\sqrt{\frac{3D}{16a\varepsilon_0\varepsilon_{env}}}$ is the plasmon frequency without the screening of interband transitions. For the interband transition whose frequency ω_n is much larger than the frequency ω where plasmon resonances appear, $D/\pi >> \varepsilon\varepsilon_0 d\omega^2$ (or $\varepsilon\varepsilon_0 d\omega\gamma_D$). Consequently, the term of $-\varepsilon\varepsilon_0 d\omega(\omega+i\gamma_D)$ in the numerator of Eq. (6) can be neglected. Therefore, $\sigma_{disk}(\omega)$ can be simplified into a form which follows the Lorentz model:

$$\sigma_{disk}(\omega)=i\frac{fS_P}{\pi}\frac{\omega}{\omega^2-\omega_p^2+i\gamma_P\omega} \qquad (7)$$

The plasmon frequency becomes $\omega_P=\frac{\omega_{P0}}{\sqrt{1+\frac{3\pi\varepsilon}{16\varepsilon_{env}}\frac{d}{a}}}$, which shows that the screening effect of interband transitions will effectively reduce the plasmon frequency for smaller disks. The plasmon damping rate is unchanged as $\gamma_P=\gamma_D$. It implies that the screening of higher energy interband transitions will not modify the damping rate. Nevertheless, in real plasmonic systems, the elastic edge scattering of carriers for smaller disks can result in the broadening of plasmon peaks. The spectral weight of the plasmon becomes:

$$S_P=\frac{D}{1+\frac{3\pi\varepsilon}{16\varepsilon_{env}}\frac{d}{a}} \qquad (8)$$

This shows that as the size of disks decreases, the spectral weight of the plasmon will reduce due to the screening of interband transitions. We apply the experimental parameters of $TaSe_2$ films (the same as those in the simulation of loss functions above) to simulate the optical conductivities of $TaSe_2$ disks. As illustrated in Supplementary Fig. 2e and 2f, when ε is set to be 4, the optical conductivity of plasmons significantly decreases as the disk radius decreases. The equivalent wave vector for disks is $q=\frac{3\pi}{8a}$, thus the screening effect on the plasmon spectral weight can be

generalized for other microstructure geometries as follows:

$$S_{\mathrm{P}} \propto \frac{1}{1+\alpha q} \qquad (9)$$

where α is a coefficient related to the intrinsic dielectric constant of interband transitions. This relation can be extended to analyze the localized plasmons in different structures beyond disks. The spectral weights of $TaSe_2$ plasmons in ribbon arrays are well fitted by Eq. (9) when the retardation effect is not pronounced.

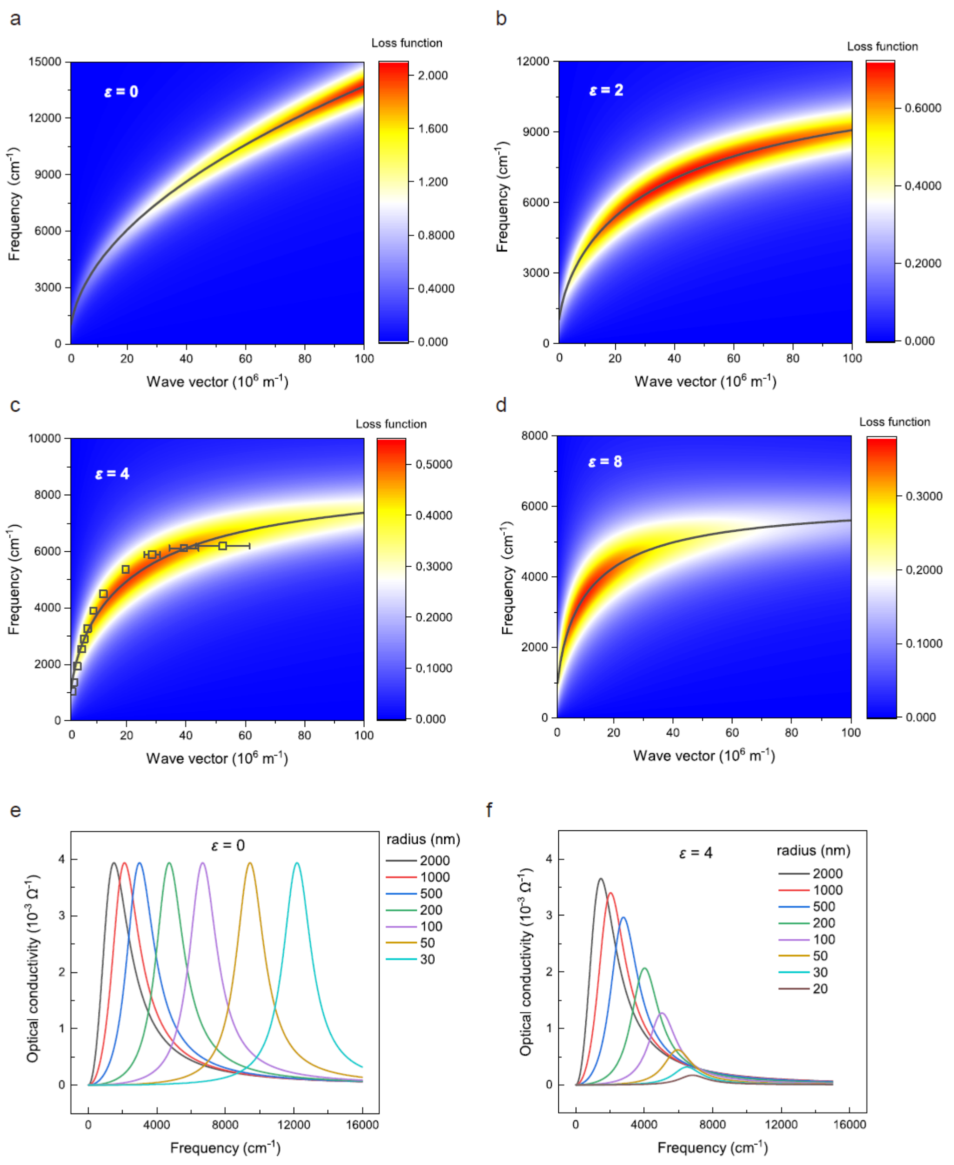

Supplementary Figure 2 Simulations of the screening effect of interband transitions on $TaSe_2$ 2D plasmons (a)-(d) The simulated loss functions of $TaSe_2$ plasmons. The intrinsic dielectric constant ε is set to be 0, 2, 4 and 8 respectively. The black solid lines denote the maximums at each wave vector. The experimental plasmon frequencies of sample T1 (d = 40 nm) are plotted in (c). **(e)** and **(f)** The simulated optical conductivities of $TaSe_2$ disks. The intrinsic dielectric constant ε is set to be 0 and 4 respectively.

Supplementary note 3: $TaSe_2$ plasmons with different film thickness

$TaSe_2$ plasmons exhibit strong layer dependence. Supplementary Figures 3a-f show the extinction spectra, the plasmon dispersion and other fitted parameters for the plasmonic devices fabricated in $TaSe_2$ films whose thickness are 25 nm (T2) and 15 nm (T3). For the 10 nm-$TaSe_2$ displayed in Fig. 3a in the main text, it is a small isolated sample, and we just fabricated one size of ribbon array to illustrate the thickness dependence. For 15 nm- and 25 nm- $TaSe_2$, the plasmon dispersion can be well fitted by Eq. (1) or Eq. (2) in the main text. Because for thinner samples, the plasmon frequency at the same wave vector is lower, thus the screening effect of interband transitions plays a less role.

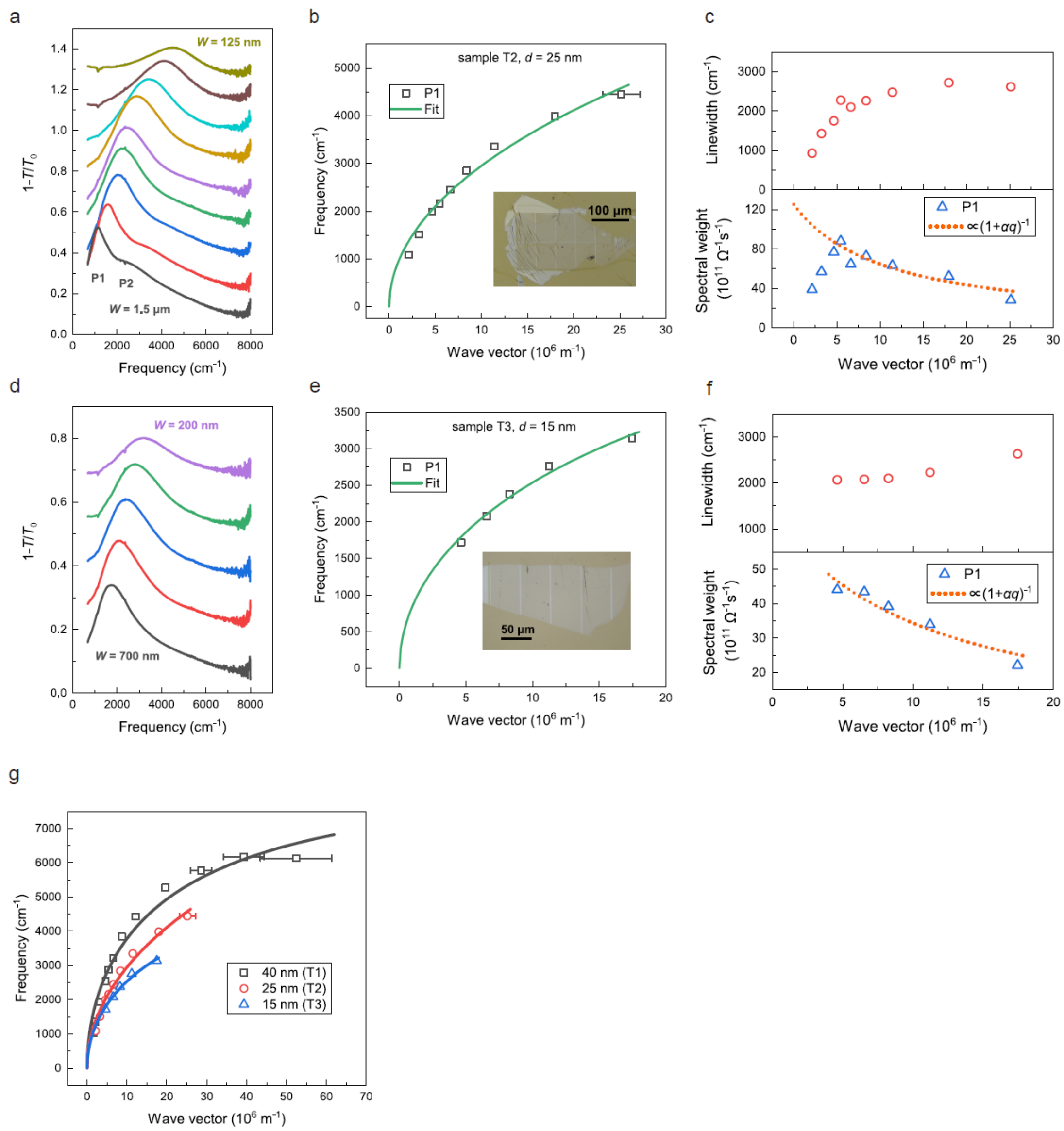

Supplementary Figure 3 The thickness dependence of 2H-$TaSe_2$ plasmons (a) The extinction spectra, **(b)** the plasmon dispersion and **(c)** the peak width and the spectral weight of plasmonic devices with thickness of 25 nm (sample T2), P1 and P2 correspond to the first and the second order modes respectively; **(d)** The extinction spectra, **(e)** the plasmon dispersion and **(f)** the peak width and the spectral weight of plasmonic devices with thickness of 15 nm (sample T3). The insets in (b) and (e) are the optical images of the corresponding $TaSe_2$ plasmonic devices, and the green solid lines are the fitted plasmon dispersion. The orange dashed lines in (c) and (f) are the fitted spectral weight at large wave vectors which follows the $S_{\mathrm{P}} \propto (1+\alpha q)^{-1}$ relation. **(g)** The

plasmon dispersions for $TaSe_2$ films with thickness of 40 nm (sample T1), 25 nm (sample T2), 15 nm (sample T3). The solid lines are the fitted plasmon dispersions by Eq. (2) in the main text.

Supplementary note 4: $TaSe_2$ plasmons in different dielectric environments

The plasmon frequency of metallic TMDCs can be manipulated by changing dielectric environments. In addition to the diamond substrate, we fabricated $TaSe_2$ plasmonic devices on BaF_2 (sample T5, d = 40 nm) and Si (sample T6, d = 40 nm) substrates. The dielectric constant ε_s of BaF_2, diamond and Si substrates at the mid-IR range are approximately 2.1, 5.7 and 11.8 respectively. As illustrated in Supplementary Fig. 4g, for the plasmonic devices with the same ribbon width and film thickness, the plasmon frequency increases as ε_s decreases. In addition, the flat plasmon dispersions on BaF_2 and Si substrates are fitted well by Eq. (2). The fitted intrinsic dielectric constant ε for these substrates is close to the results of diamond substrates. Note that, however, the maximal resonance frequency on BaF_2 is still limited by the intrinsic screening of interband transitions. The maximal plasmon frequency we observed is 6580 cm^{-1} (λ = 1.52 μm) for the plasmonic device with ribbon width 130 nm on BaF_2 substrates (Supplementary Fig. 4a).

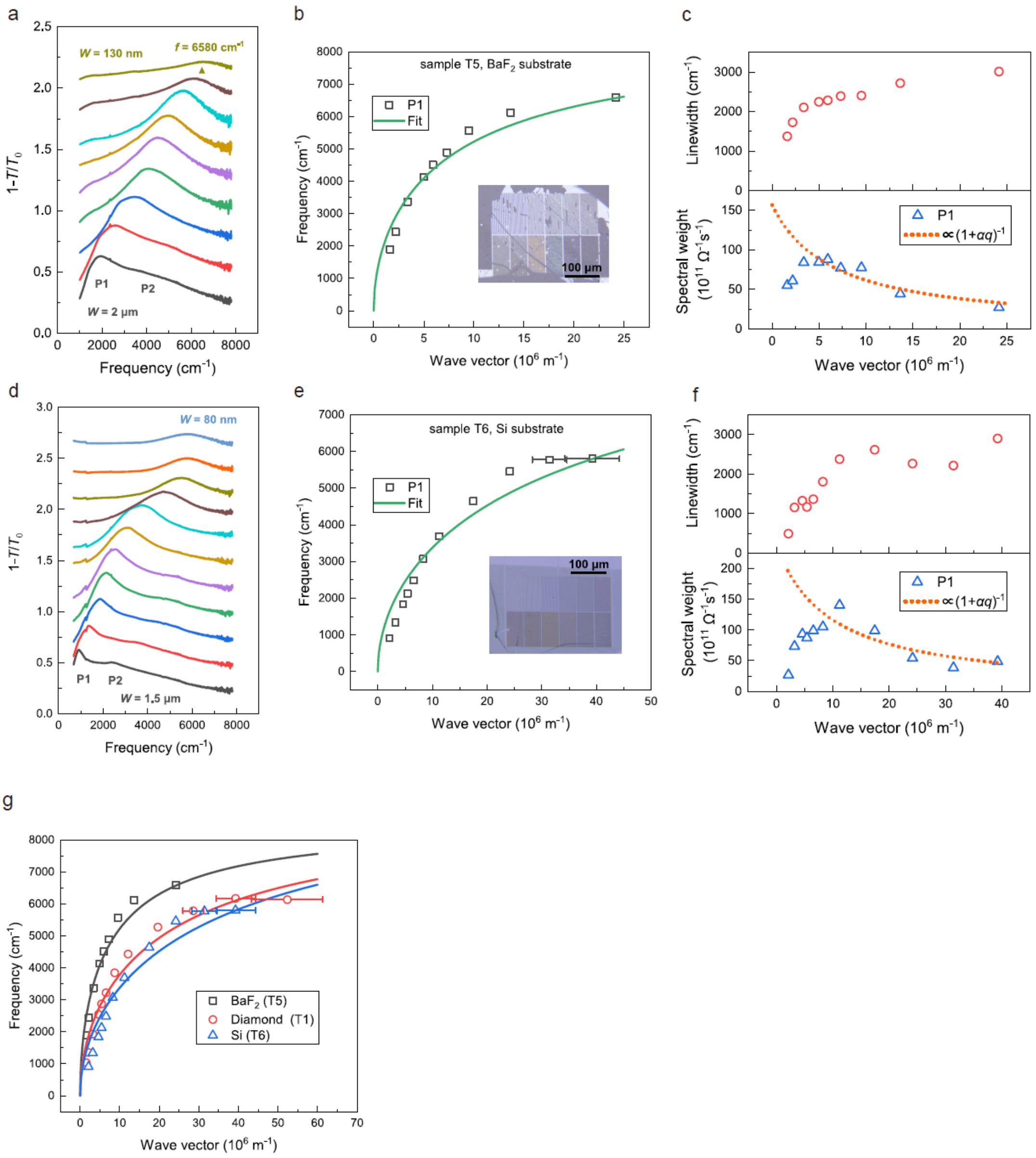

Supplementary Figure 4 $TaSe_2$ plasmons on different substrates (a) The extinction spectra, **(b)** the plasmon dispersion and **(c)** the peak width and the spectral weight of plasmonic devices on BaF_2 substrates (sample T5, d = 40 nm), P1 and P2 correspond to the first and the second order modes respectively. **(d)** The extinction spectra, **(e)** the plasmon dispersion and **(f)** the peak width and the spectral weight of plasmonic devices on Si substrates (sample T6, d = 40 nm). The insets in (b) and (e) are the optical images of corresponding $TaSe_2$ plasmonic devices, and the green solid

lines are the fitted plasmon dispersion. The orange dashed lines in (c) and (f) are the fitted spectral weight at large wave vectors which follows the $S_{\mathrm{p}} \propto (1+\alpha q)^{-1}$ relation. **(g)** The plasmon dispersions for $TaSe_2$ films on BaF_2 (sample T5), diamond (sample T1) and Si (sample T6) substrates respectively. The solid lines are the fitted plasmon dispersions by Eq. (2) in the main text.

Supplementary note 5: The temperature evolution of the intrinsic optical response and plasmons of 2H-$TaSe_2$

The intrinsic optical response of 2H-$TaSe_2$ is measured with incident light polarized parallel to the ribbon direction. As shown in supplementary Fig. 5a. It is composed of Drude response and a CDW excitation. The CDW excitation is well fitted by the Lorenz model (Eq. (2) in Supplementary note 2). Supplementary Fig. 5b shows the fitted parameters of the CDW excitation. The frequency of the CDW excitation slightly increases from 1970 cm^{-1} at 100 K to 2180 cm^{-1} at 15 K. The spectral weight prominently increases as the temperature decreases. When the polarization of incident light is perpendicular to the ribbon direction, localized plasmons are excited. Supplementary Figs. 5c-e show the temperature evolution of the fitted plasmon parameters (sample T4, d = 20 nm, W = 700 nm). Below the CDW phase transition temperature T_{C1}, the peak width of the first order plasmon prominently increases as the temperature decreases while other plasmon parameters are still in line with the original trend. The coupling rate Ω between $TaSe_2$ plasmonic and CDW excitations slightly increases from 900 cm^{-1} to 1100 cm^{-1} as the temperature decreases from 100K to 15K. For $TaSe_2$ plasmons with resonance frequency near the CDW excitation (sample T4, W = 900-400 nm, d = 20 nm), the temperature dependence is

similar. The plasmon peak width all firstly decreases as the temperature decreases and then increases as the temperature drops below T_{C1}, as illustrated in Supplementary Fig. 5f. The uncoupled plasmon peak width at 15 K is estimated from the linear fitting of peak width above T_{C1} that extrapolates to 15 K.

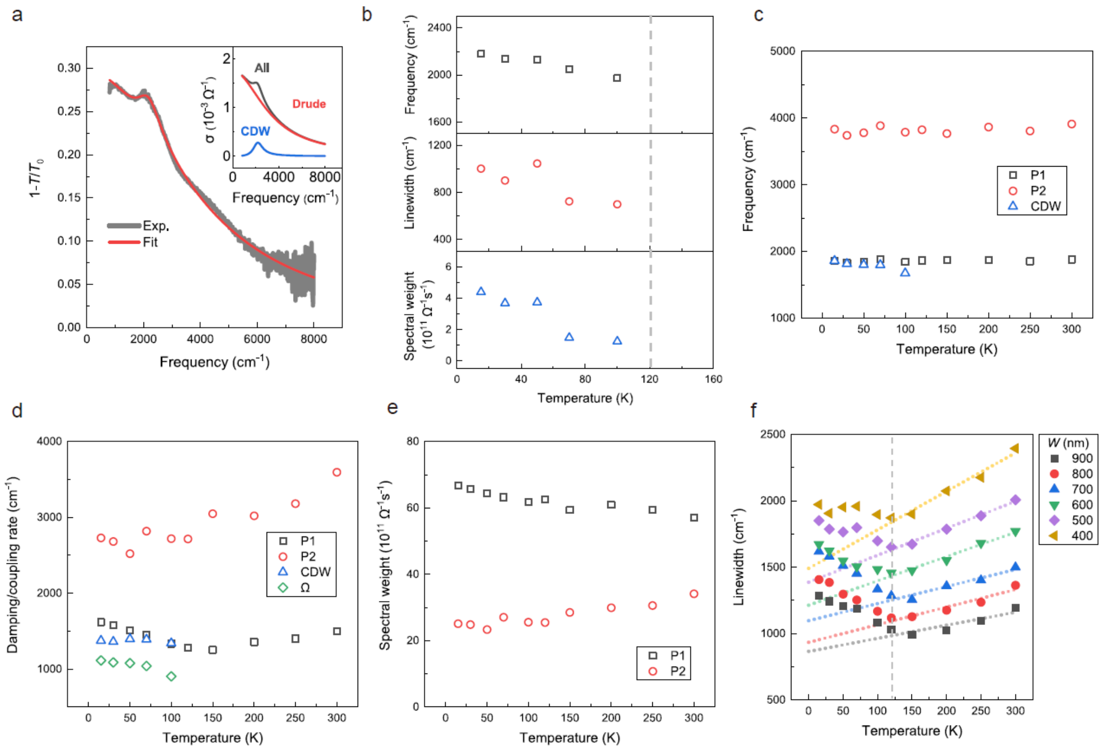

Supplementary Figure 5 The temperature-dependent intrinsic optical response and plasmons of 2H-$TaSe_2$ (a) The extinction spectrum of the intrinsic optical response of $TaSe_2$ at 15 K. The inset shows the optical conductivity decomposed into Drude response and the CDW excitation. **(b)** The spectral weight, the linewidth and the frequency of the CDW excitation at different temperature. The temperature evolution of **(c)** the frequencies, **(d)** the damping/coupling rates and **(e)** the spectral weights of the first (P1), the second (P2) order plasmon and the CDW excitation (sample T4, W = 700 nm, d = 20 nm). **(f)** The temperature evolution of the plasmon peak width for various ribbon widths (sample T4, d = 20 nm), the colored dashed lines are linear

fits for data above T_{C1}.

Supplementary note 6: The plasmon dispersion of 2H-$NbSe_2$

The plasmon resonance of 2H-$NbSe_2$ exhibits similar behaviors as 2H-$TaSe_2$ at room temperature. Because the electronic[7] and the optical properties[2-4] of them are quite close, except the appearance of CDW excitations at low temperature. As shown in Supplementary Fig. 6, the plasmon of 2H-$NbSe_2$ becomes dispersionless at large wave vectors. The fitting of plasmon dispersion by Eq. (2) in the main text gives the screening length ρ_0 of (780 ± 310) Å and the intrinsic dielectric constant ε of 3.9 ± 1.5 for a 40-nm $NbSe_2$ (sample N2).

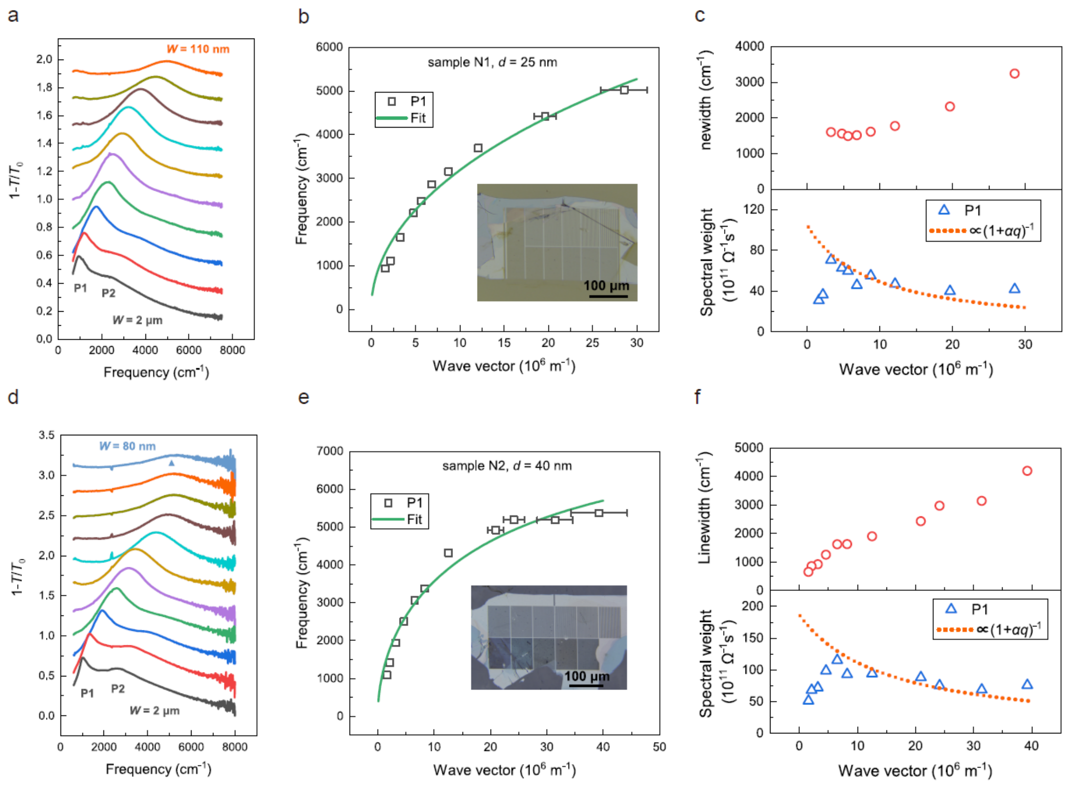

Supplementary Figure 6 The plasmons of 2H-$NbSe_2$ (a) The extinction spectra, **(b)** the plasmon dispersion and **(c)** the peak width and the spectral weight of the plasmonic devices with thickness

of 25 nm (sample N1). P1 and P2 correspond to the first and the second order modes respectively. The green line in figure (b) is the fitted dispersion by Eq. (2) in the main text, while the fitting by Eq. (1) gives similar results. **(d)** The extinction spectra, **(e)** the plasmon dispersion and **(f)** the peak width and the spectral weight for the plasmonic devices with thickness of 40 nm (sample N2). The green line is the fitted dispersion by Eq. (2) in the main text. The orange dashed lines in (c) and (f) are the fitted spectral weight at large wave vectors which follows the $S_P \propto (1+\alpha q)^{-1}$ relation.

Supplementary note 7: The temperature evolution of the intrinsic optical response and plasmons of 2H-$NbSe_2$

The plasmon peak of 2H-$NbSe_2$ continually becomes sharp as the temperature decreases. Supplementary Fig. 7a shows the temperature-dependent extinction spectra of a $NbSe_2$ plasmonic device (sample N1, W = 700 nm, d = 25 nm). The plasmon peak width prominently decreases from 1400 cm^{-1} at 300 K to 800 cm^{-1} at 5 K as illustrated in Supplementary Fig. 7b. The plasmon frequency redshifts and the spectral weight increases slightly as the temperature decreases. Other $NbSe_2$ plasmonic devices with different ribbon width or film thickness all exhibit similar temperature dependence. There is no signal of partial CDW gap at the mid-IR range (see Supplementary Fig. 7c), consistent with previous reflection measurements[4]. Supplementary Fig. 7e shows the temperature-dependent resistivity of a $NbSe_2$ thin film, the superconducting transition at $T_S \approx$ 6-7 K is observed. The kink at CDW phase transition temperature is not visible. This is probably due to the broadening of the CDW feature, which is common for the relatively thin 2H-$NbSe_2$[8].

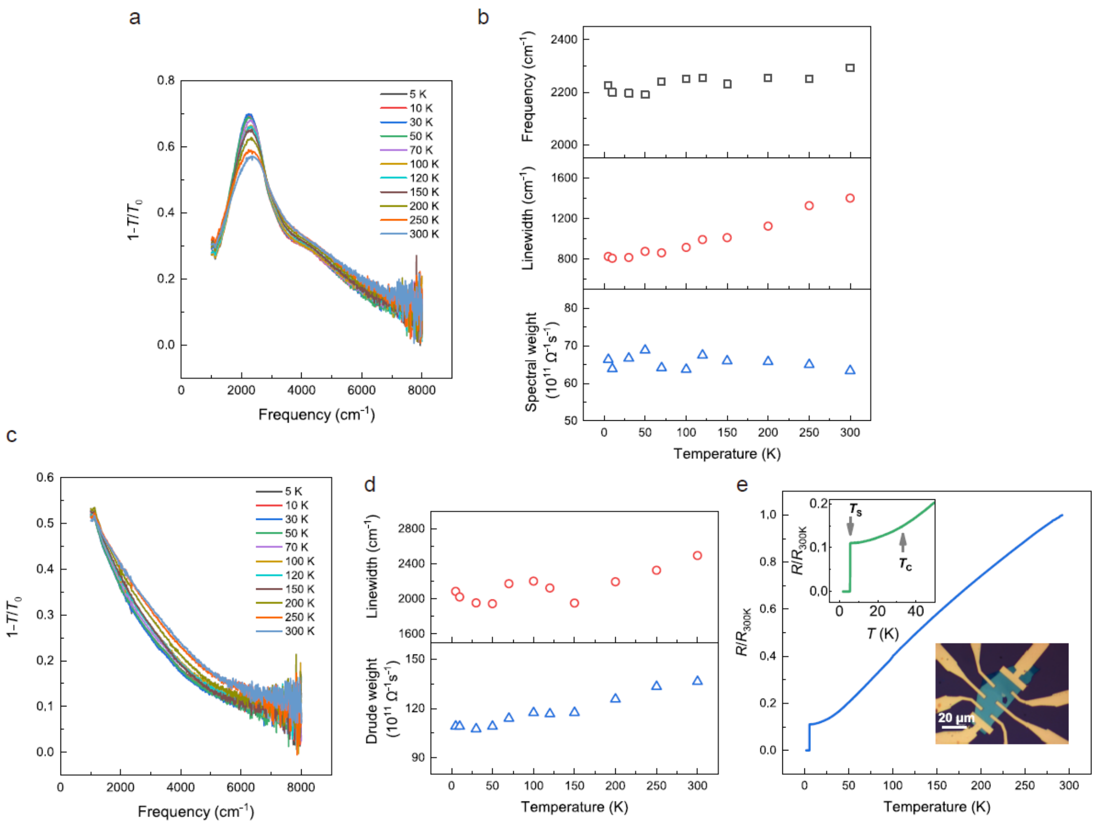

Supplementary Figure 7 The temperature evolution of the intrinsic optical response and plasmons of 2H-NbSe₂ (a) The temperature-dependent extinction spectra of a $NbSe_2$ plasmonic device (sample N1, W = 700 nm, d = 25 nm). **(b)** The spectral weight, the linewidth and the frequency of the first order plasmon peak at different temperature. **(c)** The temperature-dependent extinction spectra of $NbSe_2$ with incident light parallel to the ribbon direction. **(d)** The fitted Drude scattering rate and Drude weight at different temperature. **(e)** The normalized temperature-dependent resistivity of a 2H-$NbSe_2$ thin film. The left inset denotes the superconducting transition T_S and the possible CDW phase transition temperature T_C (≈ 33 K). The right inset shows the optical image of the $NbSe_2$ electronic device (d ≈ 20 nm)

Supplementary note 8: The coupled-oscillator model

The coupled-oscillator model can be written as follows[9]:

$$\frac{d^2x_1}{dt^2}+\gamma_1\frac{dx_1}{dt}+\omega_1{}^2x_1-\Omega^2x_2=\frac{e}{m}E(t)$$

$$\frac{d^2x_2}{dt^2}+\gamma_2\frac{dx_2}{dt}+\omega_2{}^2x_2-\Omega^2x_1=0 \qquad (10)$$

Where x_1, x_2, ω_1 and ω_2 correspond to the amplitudes and the frequencies of two oscillators respectively. γ_1 and γ_2 are their damping rates and Ω is the coupling rate. Oscillator 1 is the bright mode which can be excited by the driving electric field $E(t)=E_0e^{-i\omega t}$, while oscillator 2 represents the dark mode. The amplitudes of the two oscillators are:

$$x_1=\frac{(\omega_2^2-\omega^2-i\gamma_2\omega)}{(\omega_1^2-\omega^2-i\gamma_1\omega)(\omega_2^2-\omega^2-i\gamma_2\omega)-\Omega^4}\frac{eE(\omega)}{m}$$

$$x_2=x_1\frac{\Omega^2}{(\omega_2^2-\omega^2-i\gamma_2\omega)} \qquad (11)$$

Finally, the optical conductivity can be derived from Eq. (11) as follows:

$$\sigma_1(\omega)=-i\frac{D_1}{\pi}\frac{\omega(\omega_2^2-\omega^2-i\gamma_2\omega)}{(\omega_1^2-\omega^2-i\gamma_1\omega)(\omega_2^2-\omega^2-i\gamma_2\omega)-\Omega^4} \qquad (12)$$

Where D_1 is the spectral weight of the coupled oscillator.

We fitted the extinction spectra for samples whose plasmon frequencies are close to that of the CDW excitation (sample T4, W = 900-400 nm, d = 20 nm, T = 15 K). The fitted frequencies of the two coupled oscillators as a function of wave vectors are plotted in Supplementary Figure 8b. The plasmon frequencies in the coupled situation (T = 15 K) are close to those of in the uncoupled situation (120 K), while the frequency of the CDW excitation is at around 2000 cm^{-1}, which is also near its original frequency in the uncoupled condition. Other fitted parameters like γ_1, γ_2 and

Ω are presented and discussed in the main text (Fig. 5c and the discussion part). Due to the relatively large linewidth of these two excitations, it is challenging to observe a clear anti-crossing feature from current extinction spectra. Nevertheless, the extinction spectra can be largely modified if we can change the damping and coupling rates. Let us take the plasmonic device presented in Fig. 4 in the main text (sample T4, W = 700 nm, d = 20nm) for example. The fitted damping rates of oscillator 1 (plasmonic excitations) and oscillator 2 (CDW excitations) are γ_1 = 1618 cm^{-1} and γ_2 = 1375 cm^{-1} respectively, and the coupling rate is Ω = 1114 cm^{-1}. We calculate the extinction spectra by varying γ_2 and Ω while fixing other parameters (including those of the second order plasmon). As shown in Supplementary Figure 8c, a sharp dip emerges when γ_2 is reduced. In the limit of $\gamma_2 \ll \Omega$ and γ_1, it resembles plasmonic analogue of EIT[10]. Besides, if we continually increase the coupling rate Ω, the plasmon peak tends to split into two peaks (Supplementary Figure 8d), which behaves like Rabi splitting in the strong coupling regime[11].

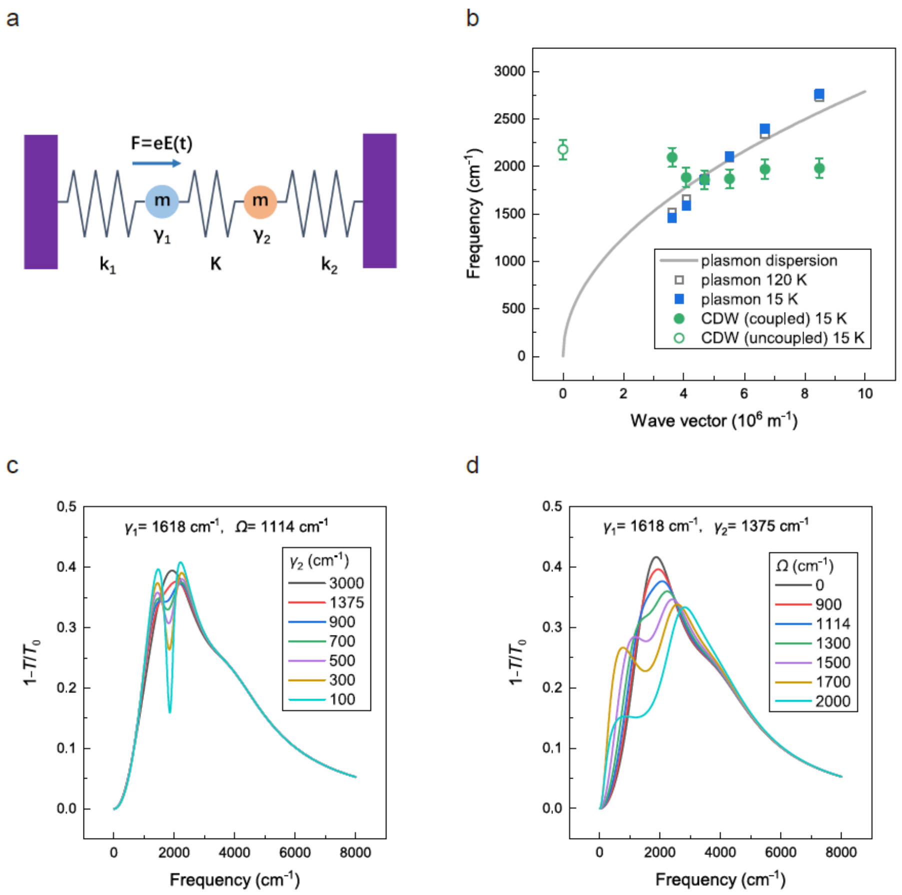

Supplementary Figure 8 The coupled-oscillator model (a) The schematic illustration of the two coupled mechanical oscillators. k_1, k_2 are spring constants and K is the coupling constant. m is the mass, $F = eE(t)$ is the driving force. **(b)** The frequencies of the plasmonic (ω_1) and the CDW (ω_2) excitations as a function of wave vectors, the grey solid line is the fitted plasmon dispersion at 120 K. The calculated extinction spectra with various **(c)** damping rate γ_2 and **(d)** coupling rate Ω.

Supplementary note 9：The Raman spectroscopy of 2H- $TaSe_2$ and $NbSe_2$ single crystals

The Raman spectra of 2H-$TaSe_2$ and 2H-$NbSe_2$ bulk single crystals were taken by a Horriba HR-Evolution2 Raman system with the excitation of a 532 nm laser (spot size ~1 μm, laser power 1 mW). The incident laser was focused by a 100 × (NA = 0.9) objective. As shown in Supplementary Fig. 9, the 2-phonon mode at 140 cm^{-1}, E_{2g} mode at 208 cm^{-1} and A_{1g} at 235 cm^{-1} are observed for 2H-$TaSe_2$, while for 2H-$NbSe_2$, the soft mode, A_{1g} mode and E_{2g} mode are at about 180 cm^{-1}, 229 cm^{-1} and 238 cm^{-1} respectively. The Raman spectra we measured are consistent with previous results[8, 12].

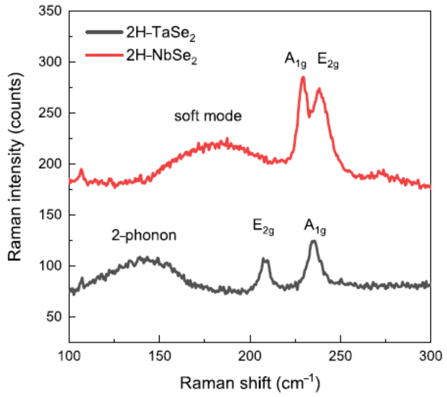

Supplementary Figure 9 The Raman spectra of 2H-$TaSe_2$ and 2H-$NbSe_2$

Supplementary note 10：The ambient stability

The thin films of metallic TMDCs exhibit good ambient stability during the period of device fabrication and optical measurements. Supplementary Fig. 10 shows the extinction spectra for one of our thinnest samples (sample T3, d = 15 nm) measured just after the fabrication and after storing for 5 months. The plasmon peak is almost the same and the small variation is within the error of measurements. During those 5 months, the plasmonic devices were measured at room temperature and low temperature several times. Each time, the devices were exposed to atmosphere for several hours. In the rest of the time, the devices were stored in glove box with inert gas environment.

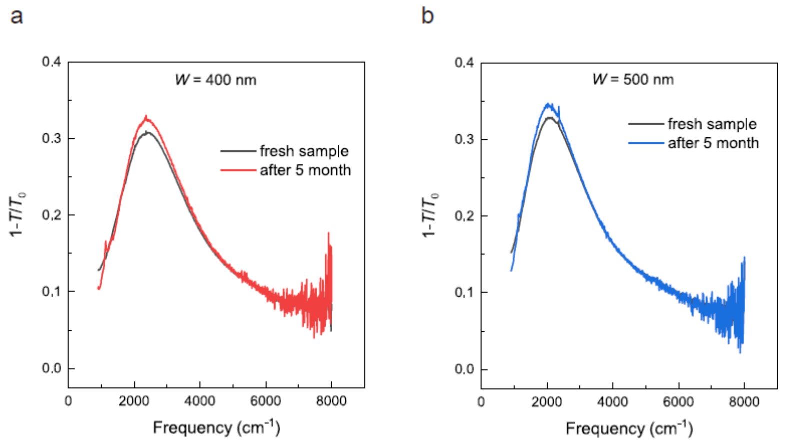

Supplementary Figure 10 The extinction spectra of plasmonic devices with ribbon width **(a)** 400 nm and **(b)** 500 nm (sample T3, d = 15 nm) measured 5 months apart.

Supplementary note 11: Carrier density determination from Hall resistance measurements

We determined the carrier density of metallic TMDCs from Hall resistance measurements. Supplementary Fig. 11 shows the Hall resistance R_{xy} as a function of magnetic field B for $TaSe_2$ and $NbSe_2$ thin films. The sheet carrier density is given by: $n_s = 1/eR_H$, where R_H is the Hall coefficient ($R_H = R_{xy}/B$), e is the elementary charge. The volume carrier density can be calculated from the sheet carrier density as $n_{3\mathrm{D}} = n_s/d$. We performed atomic force microscopy (Asylum Research MFP-3D Classic) to measure the sample thickness d. The dominant carriers for these two materials are both p-type in the normal metal state. The sheet carrier densities are 2.4 $\times 10^{17}$ cm^{-2} for the $TaSe_2$ thin film (d = 25 nm, T = 280 K) and 2.5×10^{17} cm^{-2} for the $NbSe_2$ thin film (d = 42 nm, T = 160 K). The volume carrier densities are 9.7×10^{22} cm^{-3} ($TaSe_2$) and 6.0×10^{22} cm^{-3} ($NbSe_2$) accordingly, which are comparable to previous transport results[7]. The sheet carrier density for the thin film with different thickness can be estimated from the above results assuming that $n_s \propto d$. For example, the sheet carrier density of the 40 nm-$TaSe_2$ (sample T1 described in the main text) is estimated to be 3.8×10^{17} cm^{-2}.

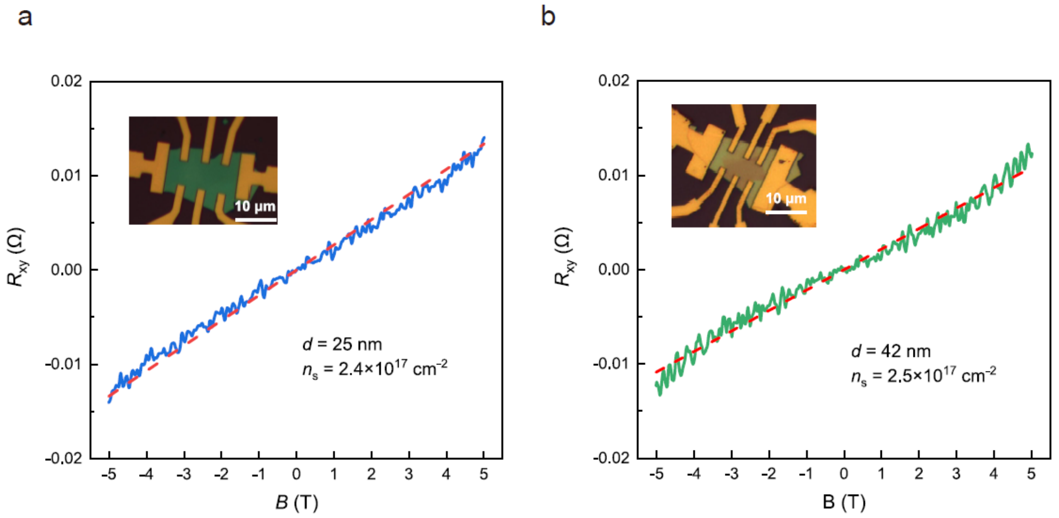

Supplementary Figure 11 Determination of charge carrier density The Hall resistance R_{xy} as a function of the magnetic field B for **(a)** a $TaSe_2$ film (d = 25 nm, T = 280 K) and **(b)** a $NbSe_2$ film (d = 42 nm, T = 160 K). The insets show the optical images of samples and the red dashed lines are linear fits to the data.

Materials	Sample number	Ribbon width (nm)	Film thickness (nm)	Substrate	Measurement Conditions
2H-$TaSe_2$	T1	60-2000 ±10	40±5	Diamond	RT, mid-IR
	T2	125-1500 ±10	25±5	Diamond	RT, mid-IR
	T3	200-700 ±10	15±5	Diamond	RT, mid-IR
	T4	400-900 ±10	20±5	Diamond	Low T, mid-IR
	T5	130-2000 ±10	40±5	BaF_2	RT, mid-IR
	T6	80-1500 ±10	40±5	Si	RT, mid-IR
	TF1	4 μm	40±5	Diamond	Low T, far-IR
	TF2	5 μm	40±5	Diamond	RT, far-IR
	TF3	7 μm	40±5	Diamond	RT, far-IR
	TF4	9 μm	40±5	Diamond	RT, far-IR
2H-$NbSe_2$	N1	110-2000 ±10	25±5	Diamond	Low T, mid-IR
	N2	80-2000 ±10	40±5	Diamond	RT, mid-IR

Supplementary Table 1 Summary of samples. For 2H-$TaSe_2$, T1-T6 are samples measured at the mid-IR range, and TF1-TF4 are samples measured at the far-IR range. N1 and N2 are 2H-$NbSe_2$ plasmonic devices. RT is for room temperature and low T is for low temperature.

Supplementary References:

1. Wang C., *et al.* Van der Waals thin films of WTe_2 for natural hyperbolic plasmonic surfaces. *Nat. Commun.* **11**, 1158 (2020).

2. Vescoli V., Degiorgi L., Berger H., Forró L. Dynamics of Correlated Two-Dimensional Materials: The 2H-$TaSe_2$ Case. *Phys. Rev. Lett.* **81**, 453-456 (1998).

3. Ruzicka B., Degiorgi L., Berger H., Gaál R., Forró L. Charge Dynamics of 2H-$TaSe_2$ along the Less-Conducting c-Axis. *Phys. Rev. Lett.* **86**, 4136-4139 (2001).

4. Dordevic S. V., Basov D. N., Dynes R. C., Bucher E. Anisotropic electrodynamics of layered metal 2H-$NbSe_2$ *Phys. Rev. B* **64**, 161103 (2001).

5. Allen S. J., Störmer H. L., Hwang J. C. M. Dimensional resonance of the two-dimensional electron gas in selectively doped GaAs/AlGaAs heterostructures. *Phys. Rev. B* **28**, 4875-4877 (1983).

6. Leavitt R. P., Little J. W. Absorption and emission of radiation by plasmons in two-dimensional electron-gas disks. *Phys. Rev. B* **34**, 2450-2457 (1986).

7. Naito M., Tanaka S. Electrical Transport Properties in 2H-NbS_2, -$NbSe_2$, -TaS_2 and -$TaSe_2$. *J. Phys. Soc. Jpn.* **51**, 219-227 (1982).

8. Xi X., *et al.* Strongly enhanced charge-density-wave order in monolayer $NbSe_2$. *Nat. Nanotechnol.* **10**, 765-769 (2015).

9. Krivenkov V., Goncharov S., Nabiev I., Rakovich Y. P. Induced Transparency in Plasmon–Exciton Nanostructures for Sensing Applications. *Laser Photonics Rev.* **13**, 1800176 (2019).

10. Liu N., *et al.* Plasmonic analogue of electromagnetically induced transparency at the Drude damping limit. *Nat. Mater.* **8**, 758-762 (2009).

11. Liu X., *et al.* Strong light–matter coupling in two-dimensional atomic crystals. *Nat. Photonics* **9**, 30-34 (2015).

12. Hajiyev P., Cong C., Qiu C., Yu T. Contrast and Raman spectroscopy study of single- and few-layered charge density wave material: 2H-$TaSe_2$. *Sci. Rep.* **3**, 2593 (2013).
